# Evaluation of two alternative non-alcohol-based media for the suspension of self-collected vaginal swabs for HPV testing in cervical cancer screening

**DOI:** 10.1016/j.heliyon.2024.e31032

**Published:** 2024-05-11

**Authors:** Chiara Giubbi, Marianna Martinelli, Maria Letizia Di Meo, Ruth Chinyere Njoku, Federica Perdoni, Robert Fruscio, Fabio Landoni, Clementina Elvezia Cocuzza

**Affiliations:** a School of Medicine and Surgery, University of Milano-Bicocca, Milan, Italy; b Fondazione IRCSS San Gerardo dei Tintori, Monza, Italy; c University of Sassari, Department of Biomedical Science, Sassari, Italy

**Keywords:** Human papillomavirus, HPV, Self-sampling, HPV testing, Cervical cancer, Suspension medium

## Abstract

The introduction of Human Papillomavirus (HPV) testing in cervical cancer screening enhanced the opportunity to introduce self-collection as an innovative approach to improve coverage rates. Validation and standardization of the pre-analytical and analytical procedures are crucial for the quality assurance of HPV tests on self-collected samples.

This study evaluated the analytical performance and the stability of self-collected vaginal samples resuspended in 5 mL of two non-alcohol-based media, eNat**®** and MSwab**®** compared to a professionally collected cervical sample, resuspended in 20 mL ThinPrep®, for the detection of high-risk HPV (hrHPV). The impact of the suspension volumes on analytical performance was also evaluated (2 and 5 ml).

A good analytical concordance in hrHPV detection in cervical and vaginal self-collected swabs suspended in 5 ml of both non-alcohol-based media was demonstrated (eNat®: 91.2 %, k = 0.821; MSwab®: 91.4 %; k = 0.798). A similar analytical performance was found for samples resuspended in 2 mL (eNat®: 92.9 %, k = 0.811; MSwab®: 92.9 %, k = 0.811) compared to cervical samples.

Good nucleic acid stability was demonstrated for vaginal samples stored at 20-25 °C and 37 °C for up to 4 weeks.

Results of this preliminary study support the introduction of these media for vaginal self-sampling-based prevention programs. Nevertheless, further research is necessary to evaluate clinical accuracy in larger settings.

## Highlights

• High-risk Human Papillomavirus (hrHPV) is the major cause of cervical cancer.
• HPV test on self-samples as strategy to implement cervical cancer screening.
• Importance of standardization of pre-analytical and analytical phases.
• Evaluation of the best combination medium/volume for suspension of vaginal samples.

## Abbreviations

HPV: Human Papillomavirus
hrHPV: high-risk Human Papillomavirus
WHO: World Health Organization
NILM: negative for intraepithelial lesion or malignancy
ASCUS: Atypical squamous cells of undetermined significance
LSIL: Low-grade squamous intraepithelial lesion
ASCH: Atypical squamous cells suspicious for HSIL
HSIL: High-grade squamous intraepithelial lesion
AGC: Atypical glandular cells
CIN1: cervical intraepithelial neoplasia grade 1
CIN2: cervical intraepithelial neoplasia grade 2
CIN3: cervical intraepithelial neoplasia grade 3

## 1 Introduction

Persistent infection of the cervical tract with high-risk Human Papillomavirus (hrHPV) has been recognized to be the major cause of cervical cancer. Cervical cancer is largely preventable through both HPV vaccination and screening for precursor lesions [1].

HPV nucleic acid testing of physician-collected cervical scrapes has demonstrated superior efficacy in reducing the incidence of cervical cancer compared to cervical cytology and its implementation in screening programs has been advocated by European guidelines [2]. Despite these advances in secondary prevention, there are still approximately 500,000 new cases of cervical cancer every year worldwide [3], occurring principally in developing countries, where women do not have access to screening programs, but also in industrialized countries in under- or never-screened women.

A promising strategy to implement cervical cancer screening programs in rural or low-resource settings as well as improving the participation rate of non-responders has been the implementation of self-collection-based screening [[4], [5], [6]]. The validation and standardization of both pre-analytical and analytical procedures of HPV testing on self-collected samples is however of fundamental importance in the quality assurance of HPV primary screening programs.

A protocol for the validation of human papillomavirus assays in combination with collections devices for HPV testing on self-samples has been published [7] allowing the evaluation of HPV test accuracy on self-samples as compared to physician-collected cervical scrapes. These studies allow to assess not only different sampling approaches using commercially available collection devices but also to extend the validation to different volumes and compositions of non-alcohol-based storage media for the resuspension of vaginal self-collected samples. Previously published validation studies of HPV tests on self-collected vaginal samples, using different vaginal collection devices, have shown great variability in the resuspension procedure used for vaginal samples, in terms of time from collection to sample processing, resuspension volume and type of liquid media being used [[8], [9], [10]].

Clinician-collected cervical samples are generally resuspended in 20 mL of ThinPrep® or PreservCyt® (Hologic, USA), an alcohol-based medium, which allows both HPV DNA molecular testing and liquid-based cytology for the triage of HPV-positive women from the same initial sample. ThinPrep® is however presently available only in 20 mL vials containing 60 % methanol, making it flammable and toxic (UN1993 Dangerous Good), with specialised handling and packaging requirements for air transportation. These aspects, together with the higher costs of alcohol-based media, represent important constraints, particularly in low-middle-income countries, making it unsuitable for vaginal sample resuspension, particularly as the latter is not an appropriate sample type for its use in cytology triage.

eNat® (Copan Italia Spa, Brescia, Italy) is a transport medium for the preservation and stabilization of nucleic acids compatible with numerous molecular assays and previously used for HPV molecular detection [11,12]. It also has the advantage of inactivating microbial agents and denaturing proteins, allowing safer sample handling in the laboratory. MSwab® medium (Copan Italia Spa, Brescia, Italy) is compatible with both bacterial and viral culture as well as nucleic acids detection. Moreover, it allows rapid nucleic acid heat extraction, thus reducing the time and costs of HPV molecular testing.

This pilot study aimed to evaluate the concordance of HPV testing of self-collected vaginal samples suspended in two alternative non-alcohol-based media, eNat® and MSwab®, using two different suspension volumes, 5 mL vs 2 mL, compared to the reference clinician-collected cervical samples suspended in 20 mL of ThinPrep®. Furthermore, viral nucleic acid stability of resuspended vaginal samples in both eNat® and MSwab® was evaluated following storage at both room temperature (RT) and 37 °C, over a timeframe of 4 weeks.

## 2 Materials and methods

### 2.1 Study design and samples collection

The study was conducted according to documentation approved by the Ethics Committee of the University of Milano-Bicocca (Protocol n. 0037320/2017 and subsequent update 0086409/2018).

A clinician-taken cervical sample and 2 self-collected vaginal swabs were obtained from 100 women referred to colposcopy at Fondazione IRCCS San Gerardo dei Tintori (Monza, Italy) following a recent abnormal cervical cytology.

Women who agreed to participate in the study by the signature of an informed consent at the time of the colposcopy visit were instructed to collect 2 vaginal swabs using FLOQSwab® 552.80 (Copan Italia Spa, Brescia, Italy), prior to gynaecological examination, while cervical swabs were taken by the physician before performing colposcopy, using L-shaped Endo/Esocervical FLOQSwab® (Copan Italia Spa, Brescia, Italy) and resuspended immediately into 20 ml of ThinPrep® (Hologic, USA). All samples, including dry vaginal swabs, were transported within 24 h from collection to the Clinical Microbiology Laboratory of the University of Milano-Bicocca, Italy.

Women underwent biopsy or conization based on the outcome of the colposcopy and on the clinical judgment. Cytological lesions were classified according to the Bethesda system [13], whilst outcomes of histological analysis according to the World Health Organization (WHO) histological classification of tumours [14].

### 2.2 Pre-analytical samples processing and HPV testing

On arrival at the laboratory, cervical samples were vortexed for 30 s and aliquots of 1.5 ml were dispensed in sterile cryotubes and stored at −20 °C until testing.

**Fig. 1:**
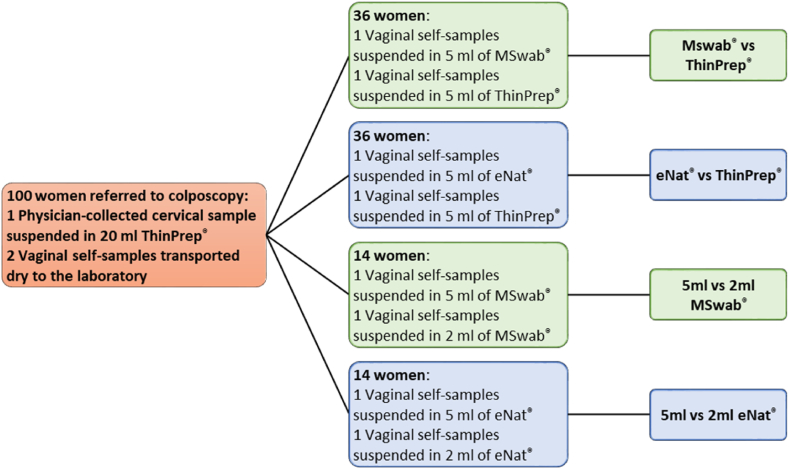
Study design.

Vaginal self-samples arrived dry at the laboratory where they were suspended in the different suspension media (ThinPrep®, eNat® and MSwab®) as described in Fig. 1. Arbitrarily, the first and second self-collected vaginal swabs were suspended respectively in the alternative non-alcohol based medium and ThinPrep® in the first two sets of 36 women. For the evaluation of the two different volumes of the alternative media, the first vaginal swab was suspended in 5 mL and the second self-sample in the 2 mL volume, respectively.

200 μl of all sample types were used to perform nucleic acid extraction using STARMag 96 × 4 Universal Cartridge Kit (Seegene, Korea) on the Nimbus platform. The automated workstation also allowed the preparation of the real-time PCR assay Anyplex™II HR HPV (Seegene, Korea). The kit was able to individually detect 14 different genotypes of hrHPV (16, 18, 31, 33, 35, 39, 45, 51, 52, 56, 58, 59, 66 and 68) and a cellular gene target by melting curves analysis. Real-time assays were performed on the CFX96 (Bio-Rad, Hercules, USA) with 5 μl of template DNA in a total volume of 20 μl. As reported by the manufacturer, if sample positivity is detected before the first cyclic Catcher Melting Temperature Analysis (cyclic-CMTA) after the first 30 cycles of amplification, the result is indicated as “+++“; if positivity is detected between the first and the second cyclic-CMTA (30 < Amplification Cycles <40) as “++“; if between the second and the third cyclic-CMTA (40 < Amplification Cycles <50) as “+“. Samples with an invalid result (no detection of the cellular gene or cellular gene detected with just “+“) were retested and excluded from the analysis if the invalidity was confirmed.

### 2.3 Evaluation of nucleic acid stability

**Fig. 2:**
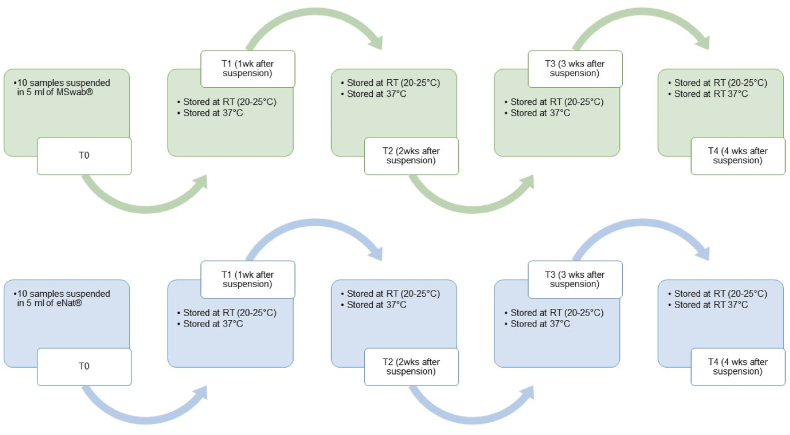
Time points and storage conditions of vaginal self-samples suspended in MSwab® and eNat® prior to testing.

In order to evaluate nucleic acid stability of 10 vaginal self-samples suspended in 5 ml of MSwab® and eNat®, aliquots of 0.4 mL of self-collected vaginal samples were dispensed in sterile cryotubes and stored at either RT (20–25 °C) and 37 °C until further testing. Nucleic acids were re-extracted starting from a new sample aliquot and hrHPV testing repeated at the time points and storage conditions described in Fig. 2.

### 2.4 Statistical analysis

Agreement between results of HPV testing of different types of samples was evaluated with Cohen's kappa (κ) statistics and defined as slight (0.00 < k < 0.20), fair (0.20 < k < 0.40), moderate (0.41 < k < 0.60), substantial (0.61 < k < 0.80) and almost perfect (0.81 < k < 1.00).

## 3 Results

### 3.1 Study population

100 women were enrolled between January 2020 and February 2022 as part of this pilot study. Three patients were excluded from the analysis: 2 of them had a reduced number of cells in the professionally collected cervical sample, resulting in an invalid HPV test result, while the other invalid result was obtained in a vaginal swab suspended in MSwab®. In total, the analysis of the results was performed on samples from 97 women (49 with one of the vaginal swabs suspended in 5 ml of MSwab® and 48 with one vaginal specimen suspended in 5 ml of eNat®).

Overall, forty-one women had a positive colposcopy examination, while in 56 no abnormalities were found. Table 1 reports the clinical data of the 97 women evaluated in this pilot study.

**Table 1:** Clinical data of patients enrolled in the study.

	n (%)
**Cytology**	**Tot. 97**
HSIL	14 (14.4)
ASCH	3 (3.1)
LSIL	36 (37.1)
ASCUS	19 (19.6)
AGC	2 (2.1)
NILM	23 (23.7)
**Biopsy/Conisation**	**Tot. 18**
Cervical cancer	1 (5.6)
CIN3	9 (50.0)
CIN2	2 (11.1)
CIN1	5 (27.7)
Negative	1 (5.6)

### 3.2 hrHPV positivity and genotypes distribution: evaluation of alternative media

Among women whose self-collected vaginal samples were suspended in 5 ml of ThinPrep® and MSwab®, 23/35 (71.4 %) cervical samples and 26/35 (74.3 %) vaginal swabs suspended in each of the two media were found to be hrHPV-positive. Among cervical samples, 8/23 (34.8 %) women had single infections and 15/23 (65.2 %) had multiple infections. 8/26 (30.8 %) vaginal specimens suspended in ThinPrep® showed single infections and 18/26 (69.2 %) multiple infections, while those suspended in MSwab® showed 7 single infections and 19 multiple infections.

Among those with vaginal self-specimens suspended in 5 ml of ThinPrep® and eNat®, the hrHPV positivity rate in cervical samples was 52.9 % (18/34), while in vaginal self-samples suspended in each of the two media was 61.8 % (21/34). The distribution of co-infections was slightly different among the three sample types: 13 single and 5 multiple infections were detected in cervical specimens, 13 single and 8 multiple infections in vaginal specimens suspended in ThinPrep® and 11 single and 10 multiple infections in vaginal swabs suspended in eNat®.

**Fig. 3:**
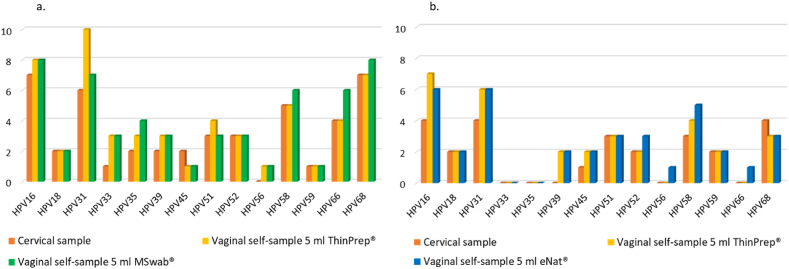
hrHPV genotypes distribution in cervical and vaginal self-samples suspended in 5 ml of ThinPrep® and 5 ml MSwab® (a) and 5 ml ThinPrep® and 5 ml of eNat® (b).

HPV16 and HPV31 were the genotypes most frequently detected. Fig. 3 and Supplementary Table 1 show the complete hrHPV genotypes distribution in cervical and vaginal self-samples in both groups of women. Among vaginal self-samples resuspended in MSwab®, 20/35 (57.1 %) samples showed the presence of the same hr-HPV types found in both the self-collected vaginal sample resuspended in ThinPrep® and in the cervical sample. A concordance for at least one hrHPV genotype among the three different types of samples was observed in 11/35 (31.4 %). On the contrary, among the 35 triplets of samples, 4 (11.4 %) have shown a discordant positive/negative result at least in one of the sample types. In 3 of these cases, the positivity found was related to just one ‘+‘, meaning that this positivity is associated with a very high Ct value ranging from 40 to 50 (Supplementary Table 1). Regarding the vaginal self-collected samples resuspended in eNat®, 24/34 (70.6 %) triplets of samples showed the presence of the same hrHPV types and 7/34 (20.6 %) demonstrated a concordance for at least one hrHPV genotype. Three triplets of samples (8.8 %) have shown a discordant result (positive/negative) and in one case the positivity found was associated with just one ‘+‘.

### 3.3 Analytical agreement between cervical and vaginal samples: evaluation of alternative media

As shown in Table 2, the concordance of hrHPV detection of cervical samples and vaginal self-collected swabs suspended in 5 ml of MSwab® and the same volume of ThinPrep® was demonstrated to be good (with k ranging from k = 0.798 to k = 0.821) irrespective of the medium used.

**Table 2:** Agreement in hrHPV detection in vaginal self-samples suspended in 5 ml of ThinPrep® and 5 ml of MSwab® (a) and 5 ml of ThinPrep® and 5 ml of eNat® (b) as compared to the reference professionally collected cervical sample.

a.	Vaginal self-sample 5 ml ThinPrep®		Vaginal self-sample 5 ml MSwab®	
	Overall concordance n (%)	k (95 % c.i.)	Overall concordance n (%)	k (95 % c.i.)
Cervical sample	31 (91.2)	0.821 (0.631–1.000)	31 (91.2)	0.821 (0.631–1.000)

b.	**Vaginal self-sample 5 ml ThinPrep®**		**Vaginal self-sample 5 ml eNat®**	
	Overall concordance n (%)	k (95 % c.i.)	Overall concordance n (%)	k (95 % c.i.)
Cervical sample	32 (91.4)	0.798 (0.583–1.000)	32 (91.4)	0.798 (0.583–1.000)

When comparing the self-collected swabs, almost no differences were obtained between samples suspended in ThinPrep® and in the alternative media (5 mL MSwab® vs 5 mL ThinPrep®: 94.3 % [33/35]; k [95 % c.i.]: 0.850 [0.650–1.000]; 5 mL eNat® vs 5 mL ThinPrep®: 100.0 % [34/34]; k [95 % c.i.]: 1.000 [1.000–1.000]).

### 3.4 hrHPV positivity and genotypes distribution: evaluation of alternative volumes

When considering the group of women whose vaginal specimens were suspended in both 2 and 5 ml of MSwab®, 10/14 (71.4 %) cervical samples and 11/14 (78.6 %) vaginal swabs suspended in the volumes of MSwab® were hrHPV-positive. Cervical specimens showed 8 single infections and 2 co-infections, while vaginal specimens suspended in 2 and 5 ml of MSwab® showed 7 single and 4 multiple infections.

In women with vaginal self-samples placed in 2 and 5 ml of eNat®, 10/14 cervical samples, 9/14 vaginal specimens suspended in 5 ml of eNat® and 11/14 vaginal swabs suspended in 2 ml of eNat® were hrHPV-positive. In the case of the two additional women whose vaginal specimen was hrHPV positive if suspended in 2 ml, but not in 5 ml of eNat®, the cervical samples were positive for HPV31 with “+” or “++” and the relative vaginal swabs suspended in 2 ml were HPV31 positive with “+“. 8 single infections and 2 multiple hrHPV infections were detected in cervical samples; 7 single infections and 4 co-infections on vaginal swabs suspended in 2 ml of eNat® and 4 single and 4 multiple infections on vaginal swabs suspended in 5 ml of eNat®.

**Fig. 4:**
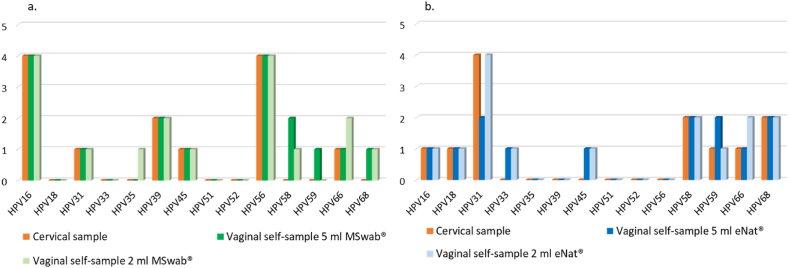
hrHPV genotypes distribution in cervical and vaginal self-samples suspended in 5 ml and 2 ml of MSwab® and 5 ml 2 ml of eNat®.

hrHPV genotypes distribution in the two groups of enrolled patients is shown in Fig. 4 and Supplementary Table 2.

### 3.5 Analytical agreement between cervical and vaginal samples: evaluation of alternative volumes

In women with vaginal specimens suspended in 2 and 5 ml of MSwab®, an almost perfect concordance was demonstrated (k = 0.811) between the cervical sample and the vaginal swabs suspended in the two different volumes.

In the group of patients with double self-collection performed in the two volumes of eNat®, the agreement was higher when 2 ml instead of 5 ml of medium was used (92.9 %, k = 8.111 vs 78.6 %, k = 0.512), probably related to low hrHPV copies number as previously described.

Data are reported in Table 3.

**Table 3:** Agreement in hrHPV detection in vaginal self-samples suspended in 5 ml and 2 ml of MSwab® (a) and 5 ml 2 ml of eNat® (b).

a.	Vaginal self-sample 2 ml MSwab®		Vaginal self-sample 5 ml MSwab®	
	Overall concordance n (%)	k (95 % c.i.)	Overall concordance n (%)	k (95 % c.i.)
Cervical sample	13 (92.9)	0.811 (0.460–1.000)	13 (92.9)	0.811 (0.460–1.000)

b.	**Vaginal self-sample 2 ml eNat®**		**Vaginal self-sample 5 ml eNat®**	
	Overall concordance n (%)	k (95 % c.i.)	Overall concordance n (%)	k (95 % c.i.)
Cervical sample	13 (92.9)	0.811 (0.460–1.000)	11 (78.6)	0.512 (0.037–0.987)

### 3.6 Stability

Good stability for up to 4 weeks was demonstrated for vaginal samples suspended in MSwab® and eNat® at room temperature (20–25 °C) and 37 °C. The results of HPV testing at the different time points and storage conditions are shown in Table 4. As reported in Table 4, the discrepancies are mainly related to a low number of viral copies with hrHPV detection with “+” using Anyplex™II HR HPV.

**Table 4:** hrHPV positivity in cervical samples and vaginal self-specimens at the different time points and storage conditions suspended in MSwab® (a) and eNat® (b).

(a)												
MSwab®	Clinical Data			Results at T0	Results after 1 week of storage		Results after 2 weeks of storage		Results after 3 weeks of storage		Results after 4 weeks of storage	
Sample ID	Colposcopy	Cytology	Histology	Vaginal self-sample	20–25 °C	37 °C	20–25 °C	37 °C	20–25 °C	37 °C	20–25 °C	37 °C
MO211	NEG	LSIL	–	33+ 59++	33+ 59++	33++ 59++	16+ 33+ 59++	33++ 59++	16+ 33+ 59++	33++ 59++	16+ 33 + 59++	33++ 59++
MO213	POS	HSIL	CIN 3	16++	16+++	16+++	16++	16++	16++	16++	16++	16++
MO214	NEG	LSIL	–	16+ 68+	68+	68+	68+	NEG	NEG	NEG	68+	68+
MO216	NEG	LSIL	–	NEG	NEG	NEG	NEG	NEG	NEG	NEG	NEG	NEG
MO14 T24	NEG	NILM	–	NEG	NEG	NEG	NEG	NEG	NEG	NEG	NEG	NEG
MO218	NEG	NILM	–	16+++ 51+++	16+++ 51+++	16+++ 51+++	16+++ 51+++	16+++ 51+++	16+++ 51+++	16+++ 51+++	16+++ 51+++	16+++ 51+++
MO219	NEG	ASCUS	–	18++ 33++	18++ 33++	18++ 33++	18++ 33++	18++ 33++	18++ 33++	18++ 33++	18++ 33++	18++ 33++
MO220	POS	ASCUS	–	31++ 66+ 68+	31++ 68+	31++ 68+	31++ 66+ 68+	31++ 66+ 68+	31++ 68+	31++ 68+	31+++ 68+	31+++ 68+
MO221	NEG	ASCUS	–	NEG	NEG	NEG	NEG	NEG	NEG	NEG	NEG	NEG
MO222	NEG	NILM	–	31+ 68+++	31+ 68++	68++	31+ 68++	31+ 68++	31+ 68++	31+ 68++	31++ 68++	31++ 68+++

(b)												
eNat®	Clinical Data			Results at T0	Results after 1 week of storage		Results after 2 weeks of storage		Results after 3 weeks of storage		Results after 4 weeks of storage	
Sample ID	Colposcopy	Cytology	Histology	Vaginal self-sample	20–25 °C	37 °C	20–25 °C	37 °C	20–25 °C	37 °C	20–25 °C	37 °C
MO235 T6	NEG	LSIL	–	16+++	16+++ 18+	16+++	16++	16+++ 18+	16+++	16+++	16++	16+++ 18+
MO269	POS	ASCUS	–	NEG	NEG	NEG	NEG	NEG	NEG	NEG	NEG	NEG
MO270	NEG	ASCUS	–	NEG	NEG	NEG	NEG	NEG	NEG	NEG	NEG	NEG
MO230 T12	NEG	NILM	–	31+ 52++	31+ 52++	31+ 52++	52++	31+ 52++	31+ 52++	31+ 52++	52++	52++
MO222 T12	NEG	NILM	–	52+ 68+++	68+++	52+ 68+++	68++	68++	68+++	68+++	68+	68+++
MO271	POS	ASCUS	–	59+	59+	59+	NEG	59+	59+	59+	NEG	59+
MO272	POS	LSIL	–	NEG	NEG	NEG	NEG	NEG	NEG	NEG	NEG	NEG
MO239 T6	NEG	NILM	–	NEG	NEG	52+	NEG	NEG	NEG	NEG	NEG	NEG
MO273	POS	HSIL	CIN 3	16++	16+++	16+++	16++	16+++	16+++	16+++	16++	16+++ 68+

## 4 Discussion

In 2021, 17 countries recommended the use of HPV tests in combination with self-sampling devices in organised screening programs, while 10 countries were conducting pilot studies on self-collected samples [15]. The introduction of self-sampling-based HPV screening programs is an opportunity to increase coverage rate by reaching non-attendant women in countries with ongoing organised screening programs, but most of all to set up new procedures in low-middle income countries where resources are limited.

In order to evaluate the impact of different suspension media and volumes on HPV test conducted on self-collected samples, we investigated the performance of two non-alcohol-based media, eNat® and MSwab®, and two suspension volumes, 5 mL vs 2 mL of each alternative medium by assessing the analytical agreement with the clinician-collected cervical sample suspended in 20 mL of ThinPrep® from the same woman.

In this study, vaginal self-collected samples were transported dry to the laboratory where they were resuspended in the evaluated media. The use of dry swabs is a suitable solution to reduce costs and avoid the risk of leakage during transport. Costs related to vaginal specimens’ management are further reduced on using non-alcohol-based media for the resuspension of the vaginal swabs and by reducing the resuspension volume compared to that used for cervical samples.

Specific protocols to evaluate the performance of HPV tests in combination with self-collection devices have been introduced [7]. Optimal sample management is a crucial step to assure sample adequacy, particularly in the case of self-collection. Technical analysis to evaluate the best workflow procedure is required, as recently underlined by Arbyn and colleagues [16]. Up to now, self-collection procedures, volumes and types of suspension media have varied across different settings and just a few studies have been conducted to evaluate the impact of pre-analytical workflow on the HPV test result on clinical samples.

The different chemical compositions of the suspension media may influence the results of the test. Some previous studies have evaluated the impact of different suspension media and volumes on HPV test results [17,18] using artificially HPV-spiked samples. Badman and colleagues tested serial dilutions of HPV-positive cell lines with 5 different suspension media and suggested that MSwab® could be considered a valid alternative to ThinPrep® [17]. The results of our preliminary analysis confirm the good analytical performance of MSwab® in HPV detection also on clinical samples and further suggest that eNat® could also be considered as a good alternative to ThinPrep®. Furthermore, in a previous published study from our group conducted on clinical specimens, the analytical performance for HPV detection on vaginal samples collected with FLOQSwab® resuspended in 5 ml eNat® was demonstrated to be comparable to that of FLOQSwab® in 5 ml ThinPrep® [19].

The potential impact of the suspension volume on the clinical performance of vaginal specimens for the detection of high-grade cervical lesions has been discussed by Inturrisi and colleagues [20] and further confirmed by Arbyn and colleagues [21]. In a more recent study, FLOQSwab® and Evalyn®Brush were suspended in different volumes of ThinPrep® spiked with various concentrations of HPV16-positive cell lines. The resuspension in a volume of 5 ml or less was demonstrated to maximize HPV detection [18]. In other studies, conducted in a colposcopy setting, paired cervical and vaginal samples were collected and suspended in different types and volumes of media underlining the effect of these factors in terms of clinical accuracy [22,23]. Furthermore, a recent study conducted by our group demonstrated a similar accuracy in HPV detection of a vaginal sample collected using FLOQSwab® suspended in 5 ml of ThinPrep® as compared to clinician-collected scrape in a group of women referred to colposcopy [24]. In the current study, a concordance of over 91 % was found between vaginal swabs suspended in 5 ml of the three tested media (ThinPrep®, MSwab® and eNat®) and cervical specimens.

Looking at the comparison of the two suspension volumes of MSwab® and eNat®, no differences were detected in the analytical performance of MSwab®, while the use of 2 ml of eNat® seemed to improve the analytical sensitivity of the method as compared to the higher 5 mL volume; however, the small sample size of women evaluated in this pilot study does not allow to make definitive conclusions. A previously published paper already evaluated the performance of 2 ml of eNat® in a screening setting [25]. Furthermore, in the present study, the discordances were detected in two women in case of a low number of copies with HPV31 detected with “+” in vaginal swabs suspended in 2 ml of the medium and no HPV detected in those suspended in 5 ml. One of the two women in whom discordances were detected had an ASCUS cytology and a negative colposcopy outcome, the other a positive colposcopy outcome and an ASCH cytology. One of the two discordances could be considered as not clinically relevant. Since the comparison was performed in a small group of women, future studies on a larger number of participants will be necessary to further investigate the potential impact of suspension volume on the analytical and clinical performance of vaginal swabs. Moreover, the data need to be better correlated with the clinical outcomes of the patients to eventually establish different cut-off values to define HPV positivity in self-collected samples as compared to cervical swabs.

Some previous studies on clinician-collected cervical samples have investigated the stability of nucleic acids in SurePath® [26] and PreservCyt® [27]. Most of the studies conducted on vaginal samples, on the contrary, focused on demonstrating the stability of dry samples to highlight the possibility of transporting samples without any suspension media to reduce costs and improve safety [28,29]. Our preliminary results on real clinical samples, using clinician-collected cervical samples as reference, indicate that MSwab® and eNat® may offer two valid alternatives for the resuspension of self-collected samples because they allow for adequate preservation of nucleic acids for up to 4 weeks at room temperature (20–25 °C) and 37 °C. The small differences across the time points are mainly related to samples with low copy numbers (detected as “+” by the assay) which are likely to result from transient infections, not significant for the development of high-grade cervical lesions. Interestingly, no invalid results were obtained at the different time points underlining the good preservation of nucleic acids in MSwab® and eNat®. Future studies using quantitative real-time PCR assay may allow a more precise evaluation of nucleic acids’ stability at the different storage conditions.

## 5 Conclusions

These preliminary results demonstrated the good performance of MSwab® and eNat® as alternative media to ThinPrep® for the suspension of vaginal specimens for HPV detection.

The main limitation of this work is the reduced sample size. Since the preliminary results are promising, future analysis will be performed to evaluate the best combination of medium/volume for suspension of vaginal samples in order to achieve an accurate and cost-effective strategy for the implementation of self-sampling in cervical cancer screening programs.

## Formatting of funding sources

This research did not receive any specific grant from funding agencies in the public, commercial, or not-for-profit sectors.

## Ethics statement

The study was conducted according to documentation approved by the Ethics Committee of the University of Milano-Bicocca (Protocol n. 0037320/2017 and subsequent update 0086409/2018). Women agreed to participate to the study by the signature of a written informed consent.

## Data availability statement

Final study datasets generated by the study are stored locally and securely at the University of Milano-Bicocca. Anonymized data will be available by request to the corresponding author on a case-by-case basis pending approval by the University of Milano-Bicocca.

## CRediT authorship contribution statement

**Chiara Giubbi:** Writing – review & editing, Writing – original draft, Methodology, Formal analysis, Data curation. **Marianna Martinelli:** Writing – review & editing, Writing – original draft, Methodology, Formal analysis, Data curation. **Maria Letizia Di Meo:** Writing – review & editing, Data curation. **Ruth Chinyere Njoku:** Writing – review & editing, Methodology, Data curation. **Federica Perdoni:** Writing – review & editing, Methodology. **Robert Fruscio:** Writing – review & editing. **Fabio Landoni:** Writing – review & editing. **Clementina Elvezia Cocuzza:** Writing – review & editing, Supervision, Project administration, Investigation.

## Declaration of competing interest

The authors declare the following financial interests/personal relationships which may be considered as potential competing interests:

Clementina Elvezia Cocuzza received research funding or gratis consumables to support research from the following commercial entities in the last 3 years: BD, Seegene, Copan, Novosanis and Hiantis.

Other authors declare that they have no known competing financial interests or personal relationships that could have appeared to influence the work reported in this paper.
