# Supplementary material for: Evaluation of two alternative non-alcohol-based media for the suspension of self-collected vaginal swabs for HPV testing in cervical cancer screening
Source: Heliyon. 2024 May 11;10(10):e31032. doi: 10.1016/j.heliyon.2024.e31032 (PMC11133750; doi:10.1016/j.heliyon.2024.e31032)
Supplement: Multimedia component 1 [file mmc1.docx]

Supplementary Table 1: hrHPV positivity in cervical samples and vaginal self-specimens suspended in 5 ml of ThinPrep^®^ and 5 ml of alternative media (MSwab® and eNat®). Analytical concordant samples for genotypes detection are highlighted in green, concordant samples for at least one genotype are highlighted in yellow. HPV genotypes detected with a discordant result with ‘+’ within the three different sample types are reported in red.

| **Sample ID** | **Clinical data** | | | **Cervical sample** | | **Vaginal self-sample 5 ml ThinPrep®** | | **Vaginal self-sample 5 ml alternative medium** | | |
| --- | --- | --- | --- | --- | --- | --- | --- | --- | --- | --- |
|  | **Colposcopy** | **Cytology** | **Histology** | **hrHPV** | **Internal Control** | **hrHPV** | **Internal Control** | **Medium** | **hrHPV** | **Internal Control** |
| MO211 | NEG | LSIL | - | 59+ | ++ | 33+  59++ | +++ | MSwab® | 33+  59++ | +++ |
| MO213 | POS | HSIL | CIN 3 | 16+++ | ++ | 16++ | +++ | MSwab® | 16++ | +++ |
| MO214 | NEG | LSIL | - | NEG | ++ | 68+ | +++ | MSwab® | 16+  68+ | +++ |
| MO216 | NEG | LSIL | - | NEG | ++ | NEG | +++ | MSwab® | NEG | +++ |
| MO14 T24 | NEG | NEG | - | NEG | ++ | NEG | +++ | MSwab® | NEG | +++ |
| MO218 | NEG | NEG | - | 16+++  51+++ | ++ | 16+++  51+++ | ++ | MSwab® | 16+++  51+++ | +++ |
| MO219 | NEG | ASCUS | - | 18++  33+ | ++ | 18++  33+ | +++ | MSwab® | 18++  33++ | ++ |
| MO220 | POS | ASCUS | - | 31++  68+ | ++ | 31++  68+ | +++ | MSwab® | 31++  66+  68+ | +++ |
| MO221 | NEG | ASCUS | - | NEG | ++ | NEG | +++ | MSwab® | NEG | +++ |
| MO222 | NEG | NEG | - | 68++ | ++ | 31+  68++ | +++ | MSwab® | 31+  68+++ | +++ |
| M0223 | POS | LSIL | CIN 2 | 16++  45++  51++ | ++ | 16++  45++  51++ | ++ | MSwab® | 16++  45++  51++ | ++ |
| M0224 | NEG | LSIL | - | NEG | ++ | NEG | ++ | MSwab® | NEG | ++ |
| M0225 | NEG | LSIL | - | 66++ | ++ | 66+++ | +++ | MSwab® | 66+++ | ++ |
| M0226 | NEG | NEG | - | NEG | ++ | NEG | +++ | MSwab® | NEG | ++ |
| M0227 | NEG | ASCUS | - | NEG | ++ | NEG | ++ | MSwab® | NEG | ++ |
| M0228 | NEG | HSIL | - | 16+  66+  68++ | ++ | 16+  66+  68++ | +++ | MSwab® | 16++  66++  68++ | +++ |
| M0229 | NEG | LSIL | - | 39++ | ++ | 39+++ | +++ | MSwab® | 39+++ | +++ |
| M0230 | NEG | LSIL | - | 31+  52++ | ++ | 31+  52++ | +++ | MSwab® | 52++ | ++ |
| M0232 | NEG | NEG | - | 58+ | ++ | 58++ | ++ | MSwab® | 58+ | ++ |
| MO043 T42 | NEG | ASCH | - | 58++ | ++ | 31+  39++  58+++ | ++ | MSwab® | 39++  58++ | +++ |
| M0319 | POS | ASCUS | - | 31+  68+ | ++ | 16++  31++  68++ | +++ | MSwab® | 16++  31++  66+  68++ | +++ |
| M0320 | NEG | LSIL | - | NEG | ++ | 31+  35++ | +++ | MSwab® | 31+  35++ | +++ |
| M0321 | NEG | LSIL | - | 16+  31++  58+++ | ++ | 16+  31++  58+++ | +++ | MSwab® | 31++  58+++ | +++ |
| M0259 | NEG | LSIL | - | 31+++  35++  52+++  58+++ | ++ | 31+++  35+++  52+++  56+++  58+++ | +++ | MSwab® | 31+++  35+++  52+++  56++  58+++ | +++ |
| MO260 | POS | LSIL | - | 45+  58++  66+++  68++ | ++ | 66++ | ++ | MSwab® | 58++  66+++  68+ | +++ |
| MO261 | POS | HSIL | CIN 1 | NEG | ++ | 31+ | +++ | MSwab® | NEG | +++ |
| MO262 | POS | ASCUS | CIN 3 | 39++  31+++ | ++ | 39++  31+++ | +++ | MSwab® | 39+++  31+++ | +++ |
| MO219 T12 | NEG | NEG | - | NEG | ++ | NEG | ++ | MSwab® | 35+ | +++ |
| MO263 | POS | LSIL | - | 16++  51++  66++ | ++ | 16+  51++  66++ | ++ | MSwab® | 16+  51++  66++ | ++ |
| MO264 | NEG | LSIL | - | NEG | ++ | NEG | ++ | MSwab® | NEG | ++ |
| MO323 | NEG | ASCUS | - | 16+++ | ++ | 16+++ | +++ | MSwab® | 16+++ | +++ |
| MO227 T12 | NEG | NEG | - | NEG | ++ | NEG | +++ | MSwab® | NEG | ++ |
| MO102 T18 | POS | LSIL | - | 18++ | ++ | 18++  33++ | +++ | MSwab® | 18++  33++ | +++ |
| MO266 | NEG | ASCUS | - | 35+  68++ | ++ | 35++  51+  58++  68+++ | +++ | MSwab® | 35++  58++  68+++ | +++ |
| MO268 | POS | LSIL | - | 52++  68+ | ++ | 52++  68++ | +++ | MSwab® | 52++  68++ | +++ |
| MO241 | POS | ASCUS | - | 16++  31++  51+++  68++ | +++ | 16++  31++  51+++  68+++ | +++ | eNat® | 16++  31++  51+++  68+++ | +++ |
| MO243 | NEG | LSIL | - | 59++ | ++ | 16+  59+ | ++ | eNat® | 59+ | +++ |
| MO244 | NEG | LSIL | - | 68+++ | ++ | 31+  68+++ | +++ | eNat® | 31+  68+++ | +++ |
| MO192 T12 | NEG | NEG | - | NEG | ++ | NEG | +++ | eNat® | NEG | +++ |
| MO245 | NEG | LSIL | - | NEG | ++ | NEG | +++ | eNat® | NEG | +++ |
| MO186 T12 | NEG | ASCUS | - | 31++ | ++ | 31+++ | +++ | eNat® | 31+++ | +++ |
| MO180 T18 | POS | LSIL | - | 51+ | ++ | 45+  51++ | +++ | eNat® | 45+  51++ | +++ |
| MO246 | POS | LSIL | - | 51+ | +++ | 51++ | +++ | eNat® | 51++ | +++ |
| MO247 | POS | HSIL | CIN 3 | 16++ | +++ | 16++ | +++ | eNat® | 16+ | ++ |
| MO034 T30 | NEG | LSIL | - | NEG | ++ | NEG | +++ | eNat® | NEG | ++ |
| MO254 | NEG | ASCUS | - | 45++  52++ | ++ | 45+++  52+++ | +++ | eNat® | 45+++  52+++ | +++ |
| MO043 T30 | POS | LSIL | CIN 1 | 31++  58++ | ++ | 16++  31+++  39++  58+++ | +++ | eNat® | 16+  31+++  39++  58+++ | +++ |
| MO228 T12 | POS | NEG | CIN 1 | NEG | ++ | 16++ | +++ | eNat® | 16++  58+  66+ | +++ |
| MO235 T6 | NEG | LSIL | - | 16++ | ++ | 16+++ | ++ | eNat® | 16+++ | ++ |
| MO269 | POS | ASCUS | - | NEG | +++ | NEG | +++ | eNat® | NEG | +++ |
| MO270 | NEG | ASCUS | - | NEG | ++ | NEG | +++ | eNat® | NEG | +++ |
| MO230 T12 | NEG | NEG | - | 52+ | ++ | 31+  52++ | +++ | eNat® | 31+  52++ | +++ |
| MO222 T12 | NEG | NEG | - | 68+ | ++ | 68+++ | ++ | eNat® | 52+  68+++ | +++ |
| MO271 | POS | ASCUS | - | 59++ | ++ | 59+ | ++ | eNat® | 59+ | ++ |
| MO272 | POS | LSIL | - | NEG | +++ | NEG | +++ | eNat® | NEG | +++ |
| MO239 T6 | NEG | NEG | - | NEG | ++ | NEG | +++ | eNat® | NEG | +++ |
| MO273 | POS | HSIL | CIN 3 | 16+++  68+ | +++ | 16++ | +++ | eNat® | 16++ | +++ |
| MO248 | NEG | AGC | - | NEG | ++ | 58++ | ++ | eNat® | 56+  58++ | +++ |
| MO249 | POS | LSIL | - | 18++ | ++ | 18+ | +++ | eNat® | 18++ | +++ |
| MO250 | POS | HSIL | CIN 2 | 31++  58++ | ++ | 31++  58+++ | +++ | eNat® | 31++  58+++ | +++ |
| MO229 T6 | POS | NEG | - | NEG | ++ | NEG | +++ | eNat® | NEG | +++ |
| MO251 | NEG | LSIL | - | NEG | ++ | NEG | ++ | eNat® | NEG | ++ |
| MO252 | NEG | LSIL | - | 18+++ | ++ | 18+++ | +++ | eNat® | 18+++ | +++ |
| MO149 T24 | NEG | NEG | - | NEG | ++ | 39+ | +++ | eNat® | 39+ | +++ |
| MO253 | POS | HSIL | CIN 3 | 58+++ | ++ | 58++ | +++ | eNat® | 58++ | ++ |
| MO256 | NEG | NEG | - | NEG | ++ | NEG | ++ | eNat® | NEG | ++ |
| MO210 T12 | NEG | NEG | - | NEG | ++ | NEG | ++ | eNat® | NEG | ++ |
| MO257 | NEG | LSIL | - | NEG | +++ | NEG | +++ | eNat® | NEG | +++ |
| MO258 | NEG | NEG | - | NEG | +++ | NEG | +++ | eNat® | NEG | +++ |

Supplementary Table 2: hrHPV positivity in cervical samples and vaginal self-specimens suspended in 5 ml and 2 ml of MSwab® and eNat®.

| **Sample ID** | **Clinical data** | | | **Cervical sample** | | **Vaginal self-sample 2 ml** | | | **Vaginal self-sample 5 ml** | | |
| --- | --- | --- | --- | --- | --- | --- | --- | --- | --- | --- | --- |
|  | **Colposcopy** | **Cytology** | **Histology** | **hrHPV** | **Internal Control** | **Medium** | **hrHPV** | **Internal Control** | **Medium** | **hrHPV** | **Internal Control** |
| MO311 | NEG | LSIL | - | 56+++ | +++ | MSwab® | 56+++ | +++ | MSwab® | 56+++ | +++ |
| MO312 | POS | ASCUS | - | 16++ | ++ | MSwab® | 16++ | +++ | MSwab® | 16++  58++ | +++ |
| MO229 T18 | NEG | NEG | - | NEG | +++ | MSwab® | NEG | +++ | MSwab® | NEG | +++ |
| MO149 T36 | NEG | NEG | - | 39+ | ++ | MSwab® | 39++ | +++ | MSwab® | 39++ | +++ |
| MO313 | POS | LSIL | - | 31+ | +++ | MSwab® | 31++ | +++ | MSwab® | 31++ | +++ |
| MO314 | NEG | LSIL | - | 39++  56+  66++ | ++ | MSwab® | 39+++  56++  66+++ | +++ | MSwab® | 39+++  56++  66++ | +++ |
| MO315 | NEG | AGC | - | NEG | ++ | MSwab® | NEG | +++ | MSwab® | NEG | +++ |
| MO286 T6 | NEG | ASCUS | - | 56++ | ++ | MSwab® | 56+++ | +++ | MSwab® | 56+++ | +++ |
| MO301 T3 | POS | HSIL | CIN 3 | 16++ | ++ | MSwab® | 16+++ | +++ | MSwab® | 16+++ | +++ |
| MO316 | POS | HSIL | CIN 3 | 16++ | ++ | MSwab® | 16++ | +++ | MSwab® | 16++ | +++ |
| MO257 T12 | POS | NEG | CIN 1 | NEG | ++ | MSwab® | NEG | +++ | MSwab® | NEG | +++ |
| MO213 T24 | POS | HSIL | CIN 3 | 16+++ | ++ | MSwab® | 16+++  66+ | +++ | MSwab® | 16+++ | +++ |
| MO318 | POS | HSIL | CIN 1 | NEG | ++ | MSwab® | 58++  35+  68++ | +++ | MSwab® | 58++  59+  68++ | +++ |
| MO322 | NEG | LSIL | - | 45+  56++ | ++ | MSwab® | 45++  56++ | +++ | MSwab® | 45++  56++ | +++ |
| MO301 | POS | HSIL | - | 16+++ | ++ | eNat® | 16+++ | +++ | eNat® | 16+  59+ | +++ |
| MO302 | POS | LSIL | - | 59+++ | ++ | eNat® | 59+++ | +++ | eNat® | 59+++ | +++ |
| MO209 T18 | NEG | NEG | - | NEG | ++ | eNat® | 33+++  45++ | +++ | eNat® | 33++  45+ | +++ |
| MO303 | POS | ASCH | NEG | NEG | ++ | eNat® | NEG | +++ | eNat® | NEG | ++ |
| MO304 | POS | ASCUS | - | 31+ | ++ | eNat® | 31+ | +++ | eNat® | NEG | +++ |
| MO305 | NEG | ASCH | - | 31++ | ++ | eNat® | 31+ | ++ | eNat® | NEG | ++ |
| MO306 | NEG | NEG | - | NEG | ++ | eNat® | NEG | +++ | eNat® | NEG | +++ |
| MO307 | POS | ASCUS | - | 31++  68++ | ++ | eNat® | 31+++  68++ | +++ | eNat® | 31+++  68++ | +++ |
| MO308 | POS | HSIL | CIN 3 | 58+++ | ++ | eNat® | 58++ | +++ | eNat® | 58++ | +++ |
| MO309 | POS | LSIL | - | NEG | ++ | eNat® | NEG | +++ | eNat® | NEG | +++ |
| MO310 | POS | LSIL | - | 68+ | ++ | eNat® | 68++ | +++ | eNat® | 68+ | +++ |
| MO250 T12 | NEG | NEG | - | 31+ | ++ | eNat® | 31++  66+ | +++ | eNat® | 31++ | +++ |
| MO317 | POS | HSIL | Cervical  cancer | 18+++  58++ | +++ | eNat® | 18++  58++ | +++ | eNat® | 18++  58++ | +++ |
| MO281 T9 | POS | ASCUS | - | 66++ | ++ | eNat® | 66+++ | ++ | eNat® | 66++ | ++ |
